# Eliciting the gender income influences on household’s food security in west africa

**DOI:** 10.1016/j.heliyon.2023.e17408

**Published:** 2023-06-22

**Authors:** Janvier Egah, Sissou Zakari, Latifou Idrissou, Néhémie Kotobiodjo, Ibrahim El Ghazi, Mohamed Nasser Baco, Marie-Paule Kestemont

**Affiliations:** a Laboratoire Société-Environnement (LaSEn), Université de Parakou, Benin; b Faculté d'Agronomie (FA)/ Université de Parakou, Benin; c Laboratoire d'Hydraulique et de Modélisation Environnementale (HydroModE-Lab), Université de Parakou, Benin; d Laboratoire de Recherche sur l'Innovation pour le Développement Agricole (LRIDA), Université de Parakou, Benin; e Institut de Statistique, de Biostatistiques et des Sciences Actuarielles (ISBA), Université Catholique de Louvain, Belgium; f Moulay Ismail University, Morocco

**Keywords:** Gender, Food security, Households, Effects, Africa

## Abstract

Women economic potential can be used to reduce household’s food insecurity in sub-Saharan Africa. This study analyzed the influence of gender on household’s food security through the household’s income in North-Benin. We selected 300 households using a multistage sampling technique. Data were collected using a questionnaire during direct interviews. Data included the households’ socioeconomic characteristics, their experiences-based Food Insecurity Scale, women and men income level. Data were analyzed using descriptive statistics and generalized structural equation modeling. The findings show that women-headed households (WHH) were less exposed to food insecurity than men-headed households (MHH). Moreover, the increasing of women income level reduced the exposure of households to food insecurity, because the increase in the income level of women stimulated the men income level. Women income also contributed more to household food expenses than men income. However, the increasing of men income level exposed the households to food insecurity. These results highlight the importance of women's empowerment in addressing household food insecurity in African’s developing countries. The findings also help policy makers to improve their knowledge for better decision making on household food security.

## 1 Introduction

Households’ food insecurity in the world has been a challenge for international community [1]. Household is food secure when it has physical, social and economic access on a permanent basis to sufficient, safe and nutritious food to satisfy their dietary needs and food preferences for an active and healthy life [2]. Food insecurity is caused by the deterioration of diet quality and favors the increasing of malnutrition forms risks [3]. Therefore, poor population were more exposed to food insecurity and to malnutrition risks.

Sub-Saharan Africa is mainly exposed to food insecurity. Approximately 22% of the population suffers from severe forms of undernourishment and food insecurity in this region [3], especially in Benin, a country in the Western Africa. Benin has an economy strongly based on agriculture which employs over 50% of the working population [4]. Agriculture contributes to 20% of Gross Domestic Product (GDP) and to 75% of exportation [4], and about 55% of the population live in the rural area among which 50% are women [5]. About 9,6% of households live in food insecurity [6]. The prevalence of severe food insecurity is higher among women than men in Benin [3]. For instance, women-headed households (WHH) (21%) are more exposed to food insecurity than men-headed households (MHH) (9%) in Benin [6]. FAO reported an increasing gender gap in food access from 2018 to 2019 [3], especially at the moderate and severe level of food insecurity. This gender gap linked with the empowerment in the households could enhance the household’s food security.

According to the theory of community food security, contextual factors and global factors can influence households [7]. The contextual factors include household’s income, household head-gender, proportion of households in food security, and the global factors include the farmer’s markets, space of shared gardening, public transportation, etc. Recent studies showed the important roles of gender and income for ensuring households food security. According to Mosha et al. [8], MHH were more food secure than WHH. In the other hand, several research showed that WHH are more food secure than MHH [[9], [10], [11]]. This contradiction rises the debate to know how the gender would contribute to household’s food security. Moreover, income level can positively influence households’ food security [11]. According to Galiè et al. [12], women carry out the income generating activities but they do not have the control of their revenue. Their priority is to spend in household’s food consumption and take care of their children, their health, etc. [13]. Thereby, women reduce men food expenditure in the households. Empowerment of women improve the households dietary diversity score [14]. Men supply households in basic food and spend the rest of their income in beverage, clothes, etc. [15]. In fact, women and men income in the households contributes differently to household’s food security, and these differences have not been recently investigated. The previous studies compared food security between WHH and MHH or analyzed the influence of household’s income on their food security [8,9,11]. Moreover, other research have analyzed the social, economic and dietary responsibilities between women and men in the households [8,10,12,16,17], but few studies have focused on the influence of gender income contribution to the intrahousehold food security. This research sets to fill the gaps and increase the knowledge on gender income contribution to the intrahousehold food security.

The present study therefore aims to analyze the influence of gender on household’s food security through incomes. This study specially addresses the contribution of contextual factors such as gender and income level to household’s food security. It hypothesizes a positive effect of women on household’s food security whereas men have a negative influence. The investigation occurred in Northern Benin. This research also analyzes the direct and indirect influences of the gender-heads on household’s food security through the gender income.

## 2 Materials and methods

### 2.1 Study area

**Fig. 1:**
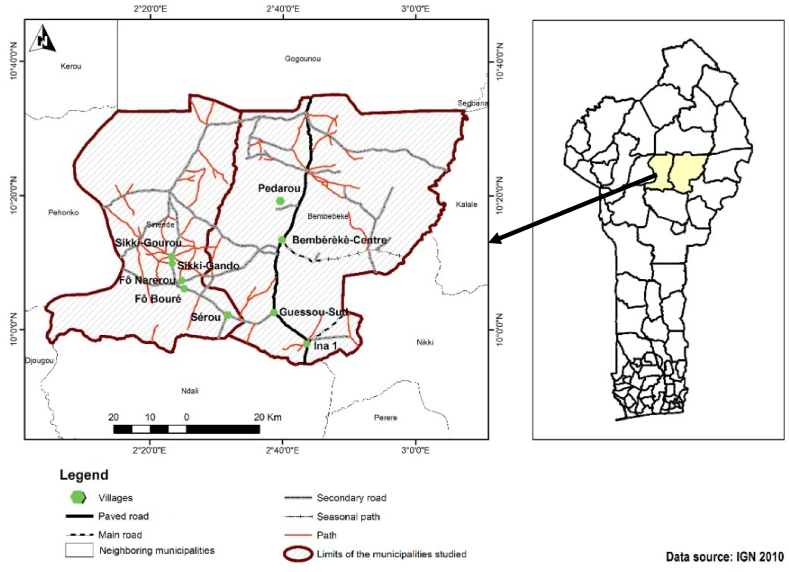
Study area in North Benin.

The study was performed in the North-Benin. The districts of Bembèrèké and Sinendé were selected for the survey because they are the transition area between cotton and food productions areas (see Fig. 1). The district of Bembèrèké has 131,255 inhabitants [5]. The majority of his population (59.2%) are rural. Women represent 49,5% of the population. About 47% of the population are married. The district of Sinendé has 91,672 inhabitants; and women represent 50.2% of the population. About 61.5% of the population of Sinendé live in rural area. About 49.8% of its population are married. The climate in the two districts is Sudano-Guinean type. Two seasons characterize these districts. The main season is the rainy season which occurs from April to October with an average annual rainfall ranged between 1000 and 1200 mm. The dry season covers from November to March. The population of the two districts cultivates cotton, maize, sorghum and soybean. They also intensively breed cattle, small ruminant and poultry.

### 2.2 Sampling design and sample size

The households were the target population of this study. The sample consisted of 300 households in the two districts (150 households per district) (Table 1). Per Cochran [18], the sample size was calculated as follow (Eq. (1)):

(1) *n* = *t*^2^(1 − *p*)/*m*^2^

where: *n* is the minimum sample size for obtaining significant results; *t* is the standard value corresponding to a confidence level (for 95% confidence level, t is 1.96); *p* is the estimated proportion of the population exhibiting the characteristic; and *m* is the Margin of error (generally set at 5%).

**Table 1:** Study villages and sample size per village.

Districts	Villages	Total respondents
Sidendé	Sikki	50
	Sérou	50
	Fô Bouré- Narerou	50
	Sous-total	**150**
Bembèrèkè	Ina 1	50
	Guessou-Sud	50
	Pedarou	50
	Sous-total	**150**
Total in study area		**300**

Three scenarios were used to obtain the sample size (Table 2). According to these scenarios, we expected a maximum sample size of 384 households. Data was collected during Covid-19 pandemic, when Benin government enjoined restrictions measures. Thus, 300 households were investigated as this size was more than the minimum requirement of the model analysis used in this study [19,20]. The households were selected using a multistage sampling technique after selection of the two districts. Firstly, three villages were selected in each district using a random sampling technique (Table 1). Then, 50 households were randomly selected per village using the systematic sampling technique [21], which consisted of enumerating and numbering households in each village [22].

**Table 2:** Scenarios of sample size estimation from different p-value.

Scenario	*p*	1-*p*	*t*	*m*	*n*
Scenario 1	0,5	0,5	1,96	0,05	384,16
Scenario 2	0,25	0,75	1,96	0,05	288,12
Scenario 3	0,125	0,88	1,96	0,05	168,07

The sampling interval (k), which is the quotient between the total number of households (N) in the village and the size of the sample (n) per village [23], was calculated with the following formula (Eq. 2):

(2) $$k=\frac{N}{n}=\frac{N}{50}$$

The sampling interval was firstly calculated in each village, then a first household was randomly selected between 1 and k. The next households were selected by adding the number of the surveyed household to the sampling interval. Besides, the individual data were collected from man and woman of each investigated household, to get information on their income level and contribution to the household’s food expenses; and we overall investigated 600 individuals, i.e. 300 households and two individual per household.

### 2.3 Data collection and analysis

Primary data were collected at two level: households and individual levels. At the households’ level, we used questionnaire during direct individual interviews to collect information on the sociodemographic and economic characteristics of households (gender of household head, his age, religion, marital status, ethnic group, education level, religion and main activity), and households experiences-based Food Insecurity Scale (HFIES) the last 12 months [2]. HFIES is a food insecurity indicator developed by FAO that measures experiences of stress, anxiety, worry and difficulties in consuming sufficient quantities of quality food based on eight dichotomic items (yes or no) over the 12 months preceding the survey [3,24,25]. HFIES was calculated as the sum of responses (yes) of all the items. Higher is HFIES, more the household is exposed to food insecurity. In individual level, the data on gender-income level in the households were collected using a questionnaire during the direct interviews. Income level was measured on a scale such as (1 = Your income is really not enough, so you need to borrow to meet expenses; 2 = Your income is not enough, so you need to use your savings to meet your expenses; 3 = Your income simply meets your expenses; 4 = Your income allows you to save a little; 5 = Your income strengthens your savings (a lot)). Data in the individual and households were collected during the same interview in April 2020. The consent of all participants to this study was acquired.

**Fig. 2:**
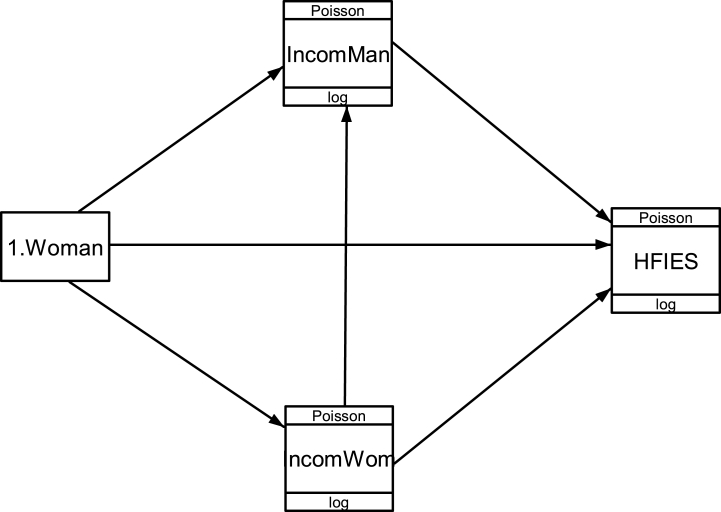
Theoretical model of generalized structural equation modeling.

Data were analyzed using the descriptive statistics and generalized structural equation modeling (GSEM) [26]. Descriptive statistics were used to characterize the socioeconomic and demographic profile of households surveyed according to the gender of household’s heads. GSEM is structural equation modeling estimation methods like a Ridge generalized least squares (RGLS) [27,28]. GSEM was used to estimate the direct and indirect effects of gender on household’s food insecurity through the women and men income levels [26]. The model was overidentified because the number of data point was 10 and the number of parameters was 6 [20]. This condition is necessary for estimating the model (See Fig. 2). The variables in the theoretical model are specified (Table 3).

**Table 3:** Description, definition and values of variables used in the theoretical model.

Type of variable	Definition	Nature	Modalities	Family/link	Sign expected	Citation
Exogenous variable	HFIES	Discontinuous variable		Poisson Log		
Endogenous variable	Gender	Dummy variable	0 = man; 1 = woman		–	Taruvinga et al. (2013) Cheteni et al. (2020) Galiè et al. (2019)
Median variable	Man income level (IncomMan)	Discontinuous variable	1 = Your income is really not enough, so you need to borrow to meet expenses; 2 = Your income is not enough, so you need to use your savings to meet your expenses; 3 = Your income simply meets your expenses; 4 = Your income allows you to save a little; 5 = Your income strengthens your savings (a lot).	Poisson Log	+	
	Woman income level (IncomWom)	Discontinuous variable		Poisson Log	–	

## 3 Results

### 3.1 Socioeconomic characteristics of surveyed households

The socioeconomic characteristics of surveyed households are presented in Table 4. Most households’ heads were men (60%). The main activity of the majority of WHH was agri-food processing (51%). Crop and animal productions were the main activities of the MHH (90%). The majority of surveyed women (75%) and of men (50%) were not educated. They belonged the same ethnic groups Bariba (84%) and had the same marital status (88%). They were in majority Muslim (65%). The surveyed women were elder than men (39 years versus 36 years). The WHH and men had the same size and number of agricultural assets. The women and MHH’ income levels were similar. However, men had a higher income level than women. The WHH and MHH had the same HFIES.

**Table 4:** Descriptive statistics of surveyed households according to gender.

		Gender of head’s household		Total (%)	Test
		Female (%)	Male (%)		
Gender		40.0	60.0	100	
Main activities	Food processing	50.8	2.3	21.7	Pearson’s Chi-square; x = 117.269; ddl = 2; p = 0.000
	Crop and animal production	32.2	89.8	66.8	
	Trade	16.9	7.9	11.5	
Education	No education	75.4	50.3	60.3	Fisher's exact test; F = 19.184; p = 0.000
	Primary education	9.3	19.8	15.6	
	Secondary education	14.4	26.0	21.4	
	University education	0.8	4.0	2.7	
Ethnic	Peulh	11.0	11.3	11.2	Fisher's exact test; F = 4.37; p = 0.216
	Bariba	83.9	84.7	84.4	
	Dendi	2.5	0.0	1.0	
	Other	2.5	4.0	3.4	
Marital status	Unmarried	4.2	13.0	9.5	Fisher's exact test; F = 18.973; p = 0.000
	Married	88.1	87.0	87.5	
	Divorcee	1.7	0.0	0.7	
	Widowed	5.9	0.0	2.4	
Religion	Traditional religion	2.5	4.5	3.7	Fisher's exact test; F = 2.305; p = 0.522
	Christian	32.2	29.4	30.5	
	Muslim	64.4	66.1	65.4	
	Other	0.8	0.0	0.3	
	Female head’s household		Male head’s household		
	Mean	Standard deviation	Mean	Standard deviation	
Age of household head	38.7	11.0	35.9	10.0	
Households size	8.0	5.5	9.3	7.9	
Agricultural assets	4.5	3.7	5.0	5.3	
Man income level	4.2	0.9	4.0	0.8	
Woman income level	3.5	1.4	3.1	1.4	
HFIES	3.2	3.4	3.1	3.1	

### 3.2 Influence of gender on household’s food security

#### 3.2.1 Effects of household’s head gender on income level and household’s food insecurity

**Fig. 3:**
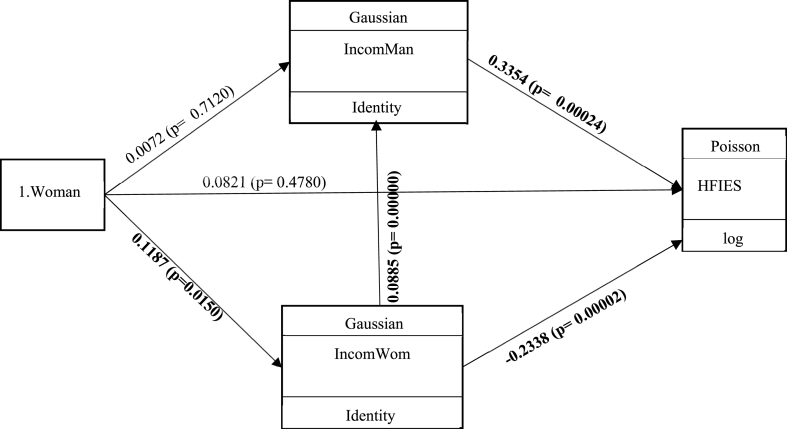
Influences of gender on HFIES using GSEM through income.

The gender of household’s heads positively influenced the women income level (p < 0.01) (Fig. 3 and Table 5). When the woman are the head households, the income level will be higher compared to MHH. Women did not provide the expenses of the extended family, but mainly care about their own children (Fig. 3 and Table 5). Furthermore, even though men carried out more income-generating activities than women; they were responsible of the expenses of all the members of their households and those of their extended family members, especially their brothers, parents, etc. These expenses could explain the positive gap of income levels between women and men.

**Table 5:** Gender and household’s food security according to GSEM (Robust test).

Variables	Coef.	Std. Err.	z	P > z	[95% Conf. Interval]	
Income level of man						
1. Female	0.0072	0.0196	0.3700	0.7120	−0.0312	0.0456
Income level of woman	0.0885	0.0080	11.0700	1,69.10-^28^	0.0729	0.1042
Constant	1.1134	0.0319	34.9300	0.0000	1.0509	1.1758
Income level of woman						
1. Female	0.1187	0.0486	2.4400	0.0150	0.0235	0.2140
Constant	1.1413	0.0326	34.9800	0.0000	1.0773	1.2052
HFIES						
1.Female	0.0821	0.1158	0.7100	0.4780	−0.1449	0.3092
Income level of man	0.3354	0.0914	3.6700	0.00024	0.1562	0.5147
Income level of woman	−0.2338	0.0549	−4.2600	0.00002	−0.3414	−0.1262
Constant	0.4733	0.3213	1.4700	0.1410	−0.1564	1.1031

The household’s head gender indirectly and negatively influenced HFIES through women’s income level (p < 0.01) (Fig. 3 and Table 5). They also control the quality and diversification of food consumed in the household (Fig. 3 and Table 5). Moreover, they access and control resources to improve their income level; thus, they purchased complementary foods, and therefore guaranteed the availability, diversification and quality of food in the household (Fig. 3 and Table 5).

#### 3.2.2 Impact of women’s income level on men income level and household’s food security

Women income level positively correlated with the men income level in the household (p < 0.01) (Fig. 3 and Table 5). Increasing the women income level by one unit enhanced the men income level by 9%. As a result, women assumed enough responsibility in the food supply of their households.

The women income level negatively influenced HFIES (p < 0.01) (Fig. 3 and Table 5). Increasing the women income level by one reduced HFIES by 23%. The increase of women income level reduced the chance of household exposure to food insecurity. The contributing foods included market garden products, meat, fish and food supplements such fruits, vegetables, etc.

Women income level had a positive indirect influence on HFIES through men income level (p < 0.01) (Table 5). Increasing the women income level indirectly raised the household’s exposure to food insecurity due to the improvement of men income level. Women income level encourages their husbands to invest in prestige expenses such as building, transport (buying cars or motorbike), etc. Men produce cotton to have cash and food crops for the household food consumption. They were insured that women must purchase the complementary food in their households.

In short, the WHH were less exposed to food insecurity than the MHH. The improvement of women income level enhanced men income level and reduced households’ exposure to food insecurity. Thus, women are the careers of society.

#### 3.2.3 Impact of men income level on household’s food security

The men income level was positively associated to HFIES (p < 0.01) (Fig. 3 and Table 5). Increasing the men income level by one increased the HFIES by 34% (Fig. 3 and Table 5). Therefore, the improvement of men income level increased the exposure of households to food insecurity.

## 4 Discussion

The study shows that the income level of woman head households was higher than the income level of MHH. This result corroborates with those of Kassie et al. [10] who reported an improvement of food security of WHH if they have similarly access and control resources as MHH, suggesting that household food security is enhanced under WHH income compared to MHH. As a result, the gender income influences the household food security, as highlighted Galiè et al. [12], Balbi et al. [17] and Mosha et al. [8] on social, economic and dietary responsibilities between women and men in the households. For Galiè et al. [12], women controlling their income provide nutritious food, money and other food to the household’s members, specifically children. Indeed, WHH are ease to access to and control the productive resources [12]. They practice food processing and other generating income activities, which contribute to improve their income level in the households [8]. Even though men carried out more income-generating activities than women; they were responsible of the expenses of all the members of their households and those of their extended family members, especially their brothers, parents, etc. These expenses could explain the positive gap of income levels between women and men.

The study shows that increasing the income level of women less exposed their households to food insecurity than the MHH. Thus, women purchased complementary foods, and therefore guaranteed the availability, diversification and quality of food in the household. These results are consistent with those of Cheteni et al. [11] in Eastern Cape Province of South Africa, where the households are more food secure when the heads is women. Moreover, women having a source of income usually contribute to household expenses which usually included breakfast for children at school, children's clothing, etc. [8]. These expenses therefore reduced the burden on men in the households. Moreover, these women had the opportunity to hire labor for their farm work; as a result, they get more time to help husbands in the farms for sowing, harvesting, etc., reducing therefore men’s expenses on labor in the farms. Thus, they lowered their production cost but enhanced their husband’s income level. Men usually controlled women income in the households, and affect some expenses of their households to women [15]. As a result, women assumed enough responsibility in the food supply of their households.

In fact, women having a high-income level contributed to foods expenses in their households, suggesting WHH were less exposed to food insecurity than MHH through increasing women’s income level. In fact, women do the housework and cooking for the households. The contributing foods included market garden products, meat, fish and food supplements such fruits, vegetables, etc. This finding corroborates with those of Cheteni et al. [11], who reported that the income positively influences the household’s food security. Otherwise, higher is the income, lower the household was likely exposed to food insecurity. The findings of this study highlighted that women income guaranteed the households food security, which confirms the results of Rogers [29] and Bonis-Profumo et al. [14]. Rogers [29] found that women expenditures are more oriented toward quality and protein-rich foods of the households. Bonis-Profumo et al. [14] showed the positive effect of empowered women on dietary diversity score. Women are responsible of the kitchen [15,29]; thus, they play an important role than men in ensuring the household food quality. Galiè et al. [12] also highlighted that women’s income improve the household nutrition more than men’s income. For Mende et al. [30], this trend can be explained by the fact that women income is often from non-agricultural activities, which give additional income and enhance household’s economy. It is the case of surveyed women whose main source of income was food processing. Thus, economic accessibility of healthy food plays an important role in households’ food and nutritional security [3].

The indirect negative effect of increasing of women income on the household’s food insecurity could explain that women income level encourages their husbands to invest in prestige expenses such as building, transport (buying cars or motorbike), etc. Men produce cotton to have cash and food crops for the household food consumption. They were insured that women must purchase the complementary food in their households. Women provide and prepare food and take care of elderly, children and sicks [13].

The exposure to food insecurity by the men income level could be due to their provision of basic foodstuffs such as maize, yams, sorghum, etc. to the households. They usually purchase land, and invest in schooling for children, buildings construction, transportation, etc. Men also seldom contributed to food diversity and did not fully cover food costs in their households, as some men use their income to marry other women and have more children for farm works, and other use their income to indulge their sexual desires in the transient rooms. Therefore, they could not provide food security for their households. The findings confirm the results of Balbi et al. [17] in Malawi and Galiè et al. [15] in Tanzania and Kenya, where men mainly spend in basic food such as maize, clothes, beverage, etc., and seldom remember the hunger of their family as women do [15]. Moreover, the main source of men income is agricultural production which fluctuates according to years [30]. In short, men income level alone could not guarantee the food security of their households.

## Conclusion and policy implication

This study shows the difference in household’s food security between WHH and MHH. The WHH mainly carry out the food processing as income generating activity. The main activity of MHH was crop and animal production. The income level of men was higher than women. However, women income level positively influenced the men income level. Increasing women income level reduce the exposure of households to food insecurity. WHH are less exposed to food insecurity than MHH. Increasing men income level raises the chance to expose the households to food insecurity. Indeed, these results confirm the present study hypotheses. These findings highlight the importance of women's empowerment in addressing household food insecurity. The decision makers are encouraged to promote women access to and control of productive resources in the households. In terms of policy implications, this study reported the influence of gender income on household’s food security. The income levels of WHH are better than those of if the MHH. The WHH are therefore less exposed to food insecurity than MHH. The findings also highlight the importance of women empowerment in rural area, and reinforce the national policy of Benin and sustainable development goals (SDG). The national plan of food security has planed the empowerment of women to reduce the household’s food insecurity. To reach SDG 2, it will be important to carry out the SDG 5 which aims to achieve and empower women. The economic empowerment of women remains also the challenges for policies in developing countries. The findings also help to improve knowledge for better decision making by policy makers in African’s developing countries.

## Declaration of competing interest

The authors declare that they have no known competing financial interests or personal relationships that could have appeared to influence the work reported in this paper.
